# A Comprehensive Evaluation of Nutritional Quality and Antioxidant Capacity of Different Chinese Eggplant Varieties Based on Multivariate Statistical Analysis

**DOI:** 10.3390/antiox14010010

**Published:** 2024-12-25

**Authors:** Jian Lyu, Ning Jin, Xianglan Ma, Xueyun Yin, Li Jin, Shuya Wang, Xuemei Xiao, Jihua Yu

**Affiliations:** 1 College of Horticulture, Gansu Agricultural University, Lanzhou 730070, China; lvjian@gsau.edu.cn (J.L.); jinn@gsau.edu.cn (N.J.); mxl202208@163.com (X.M.); xiaoxm@gsau.edu.cn (X.X.); 2 State Key Laboratory of Arid land Crop Science, Gansu Agricultural University, Lanzhou 730070, China; jinl@gsau.edu.cn (L.J.); wangsy@gsau.edu.cn (S.W.); 3 Jiuquan City Suzhou District Vegetable Technology Service Center, Jiuquan 735000, China; yxy09375910867@163.com

**Keywords:** eggplant, amino acids, polyphenols, anthocyanins, antioxidant capacity

## Abstract

Free amino acids, polyphenols, and anthocyanins were quantified in 30 Chinese eggplant varieties. Moreover, antioxidant capacity characterizations including 2, 2’-azino-bis(3-ethylbenzothiazoline-6-sulfonic acid) (ABTS), ferric-reducing antioxidant power (FRAP), and 2,2-diphenyl-1-picrylhydrazyl (DPPH) were performed. The total amino acid content of the 30 eggplant varieties ranged from 15,267.19 to 26,827.4 mg kg^−1^ DW. The most abundant amino acids were glutamic acid, arginine, and aspartic acid. The coefficients of variation (CV) for the 20 amino acids ranged from 5.85 to 106.14%, of which 18 free amino acids had CVs > 20%. Total polyphenol and anthocyanin contents ranged from 17,097.41 to 39,474.98 µg g^−1^ DW and 5.28 to 978.32 µg g^−1^ DW, respectively. The variability of both polyphenol and anthocyanin components was >20%, with a range of 21.25–102.89%. Chlorogenic acid was the most abundant polyphenol. The total anthocyanin content of purple eggplant varieties was significantly higher than green varieties. Of the purple eggplant varieties, V28 (‘E150725’), V30 (‘1952’), and V16 (‘Weichangqie101’) had significantly higher total anthocyanins than the other eggplant varieties. DPPH, ABTS, and FRAP assays showed peaks at V3 (‘Zhengqie924’). Pearson’s correlation analysis revealed that polyphenols and anthocyanins were the main contributors to the antioxidant capacity of eggplants. A classification model with principal component analysis classified 30 Chinese eggplant varieties into two categories: high and low antioxidant capacities. The top five Chinese eggplant varieties ranked for amino acids, antioxidants, and antioxidant capacity were V29 (‘Zhengqie903’), V24 (‘Zhengqie78’), V1 (‘1871’), V3 (‘Zhengqie924’), and V28 (‘E150725’). These findings provide theoretical basis for high-quality breeding and producer/consumer selection of eggplants.

## 1. Introduction

Eggplant (*Solanum melongena* L.) is one of the most widely grown vegetables in the world and the sixth most abundant vegetable globally after tomato, watermelon, onion, cucumber, and cabbage [1]. It is also the third most consumed fruit in the family Solanaceae, with only a few reports on the use of eggplant leaves as an herb or for medicinal purposes. Eggplants originated from Southeast Asia, and a large proportion of world production is highly concentrated in Asia and the Mediterranean Basin, and the highest production and consumption is in China [2]. Vegetables and fruits, with their basic and essential nutritional requirements for humans, are an important part of the daily diet. Many human diseases are caused by an unbalanced diet or insufficient nutrition. A balanced diet is essential for human health [3]. Consequently, there has been an increasing demand for attention to be given to the potential nutrient components of fruits and vegetables. Considerable research has focused on identifying the primary and secondary metabolisms of vegetables and fruits, with the aim of breeding varieties with favorable properties for human health to improve diets and combat malnutrition [3,4].

Vegetables and fruits are reservoirs for countless bioactive compounds. Epidemiological studies have shown that antioxidant compounds derived from vegetables and fruits can dramatically reduce mortality rates caused by heart disease, cancer, other degenerative diseases, and aging [5]. More than 5000 phytochemicals have been isolated and characterized from fruits, vegetables, and grains [6]. Among dietary phytochemicals, phenolic compounds have been the most investigated. Eggplants are nutrient-rich because of the presence of carbohydrates, vitamins, proteins, minerals, and bioactive compounds [7]. Secondary metabolites in eggplant fruits, particularly polyphenol (including phenolic acids and flavonoids) components, are considered a promising source of antioxidants [8]. Phenolic acids are abundant in the plant kingdom and have beneficial properties for health benefits. In particular, hydroxycinnamic acid derivatives have promising effects in treating cardiovascular diseases (CVD), obesity, and type 2 diabetes [9]. The remarkable anti-herpetic activity of eggplant peel extract (EPE) was highlighted in a study aimed at evaluating the health properties of the extract, which was associated with a significant increase in certain flavonoids [10]. Anthocyanins are natural pigments in plants with a wide range of colors, covering almost the entire visible spectrum, from red to purple to blue hues [11]. Consumption of anthocyanins has been reported to have several favorable roles in human health, mainly including mitigating effects on cancer, inflammation, and CVD [12]. Six basic types of anthocyanin structures are found in plants, namely pelargonidin, cyanidin, delphinidin, peonidin, petunidin, and malvidin [13]. To date, >500 anthocyanins have been isolated from plants, including eggplant [11]. Delphinidin derivatives are the only anthocyanins identified in eggplant fruit [8], and delphinidin-3-rutinoside is the main anthocyanin in eggplant peel [14]. Eggplant is classified as one of the principal vegetables with high antioxidant capacity owing to its outstandingly high profile of anthocyanins and phenolic compounds; hence, eggplants can be used to prepare different functional foods with beneficial properties [15]. Horincar et al. [16] evaluated the efficacy of adding EPE to the beer-brewing process. Beers formulated with EPE showed higher anthocyanin, flavonoid (catechins), and phenolic compound (gallic acid) contents than those in controls without EPE. EPE addition to beer increased the antioxidant activity of the final product (free radical scavenging by 2,2-diphenyl-1-picrylhydrazyl (DPPH) and 2, 2’-azino-bis(3-ethylbenzothiazoline-6-sulfonic acid) (ABTS)). In addition, beers containing EPE exhibited a significantly higher reddish hue owing to the higher anthocyanin content of EPE.

Eggplants have been cultivated for centuries, and their value as natural sources of antioxidants is now being recognized. Secondary metabolites in eggplant fruits, particularly phenolic compounds, have been studied extensively. However, certain primary metabolites that contribute to nutritional value and dietary quality have been neglected. Primary metabolites (including sugars, vitamins, amino acids, organic acids, lipids, and nucleotides) are essential fruit metabolites that play an indispensable role in plant development, fruit flavor, and aroma [17]. Amino acids are the primary metabolites and basic units of proteins and have many prominent functions. They provide a foundation for several biosynthetic pathways and play essential roles in signal transduction and plant stress responses [18]. Eight essential amino acids (i.e., lysine, tryptophan, phenylalanine, methionine, threonine, isoleucine, leucine, and valine) are obtained from daily foods to maintain normal body function. Glutamine, threonine, and lysine are typically used to maintain intestinal tract integrity and health [19]. In addition to its medicinal value, glutamine (monosodium glutamate) is used as a food flavoring agent [20]. Aspartic acid is a precursor substance necessary for purine, pyrimidine, asparagine, and inositol synthesis [21]. Some amino acids in various foods (e.g., glutamate and aspartic acid) also scavenge free radicals and thus have antioxidant properties [22]. Some amino acids are closely associated with human taste. Alanine, glycine, and serine are more favorable for a sweet taste, whereas, leucine, phenylalanine, tryptophan and tyrosine mainly contribute to a bitter taste [23].

Nutrient content is an essential consideration when selecting eggplant varieties for cultivation. The levels of carbohydrates, proteins, total phenolics, anthocyanins, flavonoids, chlorogenic acids, and antioxidant capacity of 13 commercial eggplant varieties grown locally in Greece were evaluated [24]. Another study was conducted to evaluate the phenolic acids, anthocyanins, and antioxidant potential of four different varieties of eggplants (long green, purple large, purple medium, and purple small) [25]. These findings make a contribution to the understanding of the metabolic composition of local eggplant bioactive components and the diversity of their genetic backgrounds and provide a theoretical basis for the development of breeding strategies to improve eggplant fruit quality. Nevertheless, all these studies were evaluation studies on eggplant varieties from other countries and had smaller sample sizes. Therefore, the present study aimed to clarify the diversity of amino acids and polyphenols in 30 eggplant varieties widely grown in China to provide a theoretical basis for development of future eggplant crop improvement strategies and consumer preference choices.

## 2. Materials and Methods

### 2.1. Plant Materials

The 30 eggplant varieties selected for the experiment were planted on 15 September 2021 and harvested at commercial maturity stage on 20 December 2021 in the Gobi Agricultural Park, Zongzhai Town, Suzhou District, Jiuquan City, Gansu Province (39°39′22″ N, 98°40′0″ E). The region has a temperate continental arid climate. The varieties were grown in solar greenhouses using substrate cultivation. The area of each variety plot was 80 m^2^ (size: 10 × 8 m). Irrigation and fertilization of eggplants were carried out using water–fertilizer integration. All eggplant varieties were grown under the same conditions and daily management practices. Eighteen eggplant fruits (three replicates of each variety; six eggplants per replicate) of the same size, of similar maturity, and that were free from pests and diseases were selected for analysis. The numbers, individual names, source details (e.g., company and series), and agronomic traits are provided in Table S1. Eggplants were chopped, mixed well, and frozen in liquid nitrogen at −80 °C for subsequent experiments.

### 2.2. Chemicals

The 20 free amino acid standards used for quantitative analysis were obtained from Merck and Sigma-Aldrich (St. Louis, MO, USA). The hydrochloric acid used for amino acid extraction was an analytically pure reagent. The mobile phases (ammonium formate and acetonitrile) used for the determination of amino acids by high-performance liquid chromatography coupled to mass spectrometry (LC-MS) were chromatographically pure reagents. Compounds were identified based on the retention times of polyphenol and anthocyanin standards (both purchased from Yuanye Biotechnology Co., Ltd., Shanghai, China) and quantified according to a standard curve. The methanol and acetic acid used in the experiments for polyphenol and anthocyanin extraction and determination were chromatographically pure reagents. Oxalic acid, zinc sulfate, potassium ferricyanide, and 2,6-dichloroindophenol sodium salt used for vitamin C (VC) extraction and determination were analytically pure reagents.

### 2.3. Determination of Amino Acid Components

Eggplants were frozen in liquid nitrogen, dried in a freeze dryer (LyoQuest-85, Telstar Technologies, Barcelona, Spain) for 72 h, and then milled in a grinder (TissueLyser II; QIAGEN, Hilden, Germany). Samples (0.1 g) with 1 mL of 0.5 M hydrochloric acid solution were centrifuged for 20 min at 20,000× *g* (3–18 KS; Sigma, Osterode am Harz, Germany) after sonication. An injection of 5 µL of the supernatant obtained by passing through a 0.22 µm aqueous filter was performed in an LC-MS (Agilent 1290-6460, Agilent Technologies Inc., Santa Clara, CA, USA) for quantitative analysis. The HPLC parameters used were as follows: mobile phase A was water/200 mM ammonium formate stock solution = 9:1; mobile phase B was acetonitrile/ammonium formate stock solution = 9:1. The final concentration of both mobile phases A and B was 20 mM. The mobile phase was set to a 0.5 mL/min flow rate. The column was an Agilent InfinityLab Poroshell 120 HILIC-Z (2.1 × 100 mm, 2.7 µm) at 25 °C. The mass spectrometry source conditions were as follows: acquisition mode of the multi-response monitoring model; the ionization mode was electrospray ionization (ESI) and was performed in the positive ion mode; the dry temperature was set to 330 °C; the atomizer pressure remained at 35 psi; the gas flow rate was controlled at 13.0 L·min^−1^; the sheath temperature was set to 390 °C; the sheath gas flow rate remained at 12 L·min^−1^; and the capillary voltage was maintained at 1500 V.

### 2.4. Determination of Polyphenol and Anthocyanin Components

Polyphenols and anthocyanins components were extracted and determined according to the approach of Jin et al. [26] with minor modifications. Freeze-dried eggplant powder (0.1 g) and 2 mL of 90% (*v*/*v*) methanol were extracted by ultrasonication in a water bath at 50 °C for 20 min. Samples were then centrifuged at 4 °C and 8000 rpm for 10 min, and the supernatant was collected for analysis. After filtration using a 0.22 µm organic phase filter membrane, the supernatant (10 µL) was analyzed by HPLC using a ZORBAX SB-C18 column (250 × 4.6 mm, 5 µm, Waters Corp., Milford, MA, USA). The mobile phases were methanol (A) and 1% (*v*/*v*) acetic acid (B), and the column temperature was maintained at 30 °C. The mobile phase flow rate was controlled at 1.1 mL min^−1^. Compounds were detected at 280 nm (gallic and benzoic acids), 322 nm (gentianic acid, chlorogenic acid, caffeic acid, cynarin, p-coumaric acid, ferulic acid, delphinidin chloride, and rutin), and 520 nm (delphinidin-3-O-rutinoside chloride).

### 2.5. Determination of Vitamin C Content

The VC content of eggplant fruits was determined using the 2, 6-dichloroindophenol staining method. Fresh eggplant fruit samples (0.5 g) and 1.5 mL of 2% oxalic acid were ground until homogenized, and the mortar was rinsed with 10 mL of 1% oxalic acid. The homogenate was transferred to a 50 mL volumetric flask, and 0.5 mL of 30% zinc sulfate was added, followed by shaking and the addition of 0.5 mL of 15% potassium ferricyanide. Finally, 1% oxalic acid was added to a final volume of 50 mL, and the solution was filtered. Filtrate (4 mL) was placed into a test tube, 2 mL of 2,6-dichloroindophenol sodium salt solution and 5 mL of xylene were sequentially added, and the solution was shaken well and allowed to stratify. Absorbance of the upper solution was measured at 500 nm and zeroed with xylene.

### 2.6. Determination of Antioxidant Parameters

The total antioxidant capacity of eggplant fruits was measured according to the kit instructions (Sino Best Biological Technology Co., Ltd., Shanghai, China), covering ABTS radical scavenging activity, ferric-reducing antioxidant power (FRAP), and DPPH radical scavenging activity. Absorbance at 734, 593, and 515 nm was measured using a UV-1800 visible spectrophotometer following the manufacturer’s protocol (Shimadzu, Kyoto, Japan), where ABTS and DPPH are in %, and FRAP is in U g^−1^ fresh weight. The antioxidant capacity of eggplant fruits was quantified by the change in absorbance. VC was used as the control.

### 2.7. Statistical Analysis

Data were analyzed using one-way analysis of variance in SPSS software (version 22.0; SPSS Institute Inc., Chicago, IL, USA), and significant differences were compared using Duncan’s multiple range test (*p* < 0.05). Boxplots were constructed using a free online data analysis platform on the MetaboAnalyst 5.0 server (accessed 22 November 2023; https://cloud.metware.cn). Correlation analysis and principal component analysis (PCA) were performed using Origin 2021 software (Origin Inc., San Francisco, CA, USA). Results are presented as the mean value ± standard error. In all analyses, a *p*-value < 0.05 was considered statistically significant. To further evaluate the differences in amino acids, antioxidant substances, and antioxidant capacity of fruits of different Chinese eggplant varieties. The scores of each component were automatically calculated based on the matrix and standardized data using Origin 2021 software. The PCA-based total scores were weighted by the variance contribution of each principal component, and the calculation formula was as follows:

(1) $$Q_{i}=\sum_{m=1}^{8}P_{m}Z_{im}$$

where *Q* indicates the total score; *i* indicates the amino acids, antioxidant substances, and antioxidant capacity of the *i*th of the V*_i_*; *m* denotes the m principal components; *P_m_* is the contribution of the m principal components to each other’s variance; and *Z_im_* is the score of the m principal components of the amino acids, antioxidant substances, and antioxidant capacity of the V*_i_*.

## 3. Results

### 3.1. Difference Analysis of Amino Acids

#### 3.1.1. Variance and Diversity Analyses of Amino Acids

The content of 20 amino acids in 30 eggplant varieties was determined using LC-MS. Variance analysis showed significant differences in the content and composition of free amino acids in the different eggplant varieties (Table S2). The top five eggplant varieties with the highest free total amino acid content were V21, V29, V1, V17, and V26; the top five eggplant varieties with the lowest free total amino acid content were V20, V5, V4, V2, and V14. Diversity analysis showed that the top three amino acids among the 30 eggplant varieties were glutamic acid, arginine, and aspartic acid (Table 1). The standard deviation of the 20 amino acids of the different eggplant varieties ranged from 0.76 to 1883.77, and the coefficient of variation (CV) ranged from 5.85 to 106.14% (Table 1).

#### 3.1.2. Correlation Analysis and PCA of Amino Acids

**Figure 1:**
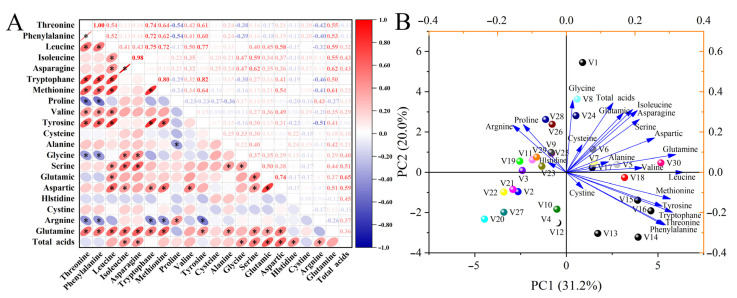
(**A**) Pearson’s correlation analysis and (**B**) principal component analysis of amino acids in different eggplant varieties. Data are mean values (*n* = 3). * indicates a significant correlation (*p* < 0.05) (two-tailed). V1–V30 represent 30 different Chinese eggplant varieties.

Pearson’s correlation analysis revealed significant positive and negative correlations among the 20 amino acid components of the 30 eggplant varieties (Figure 1A). Aspartate was positively correlated with leucine (r = 0.58), isoleucine (r = 0.37), tryptophan (r = 0.54), valine (r = 0.49), serine (r = 0.38), and glutamate (r = 0.74). Significant positive correlations were also observed between glutamate and leucine (r = 0.45) and serine (r = 0.50). Phenylalanine was significantly positively correlated with glutamine (r = 0.53), tyrosine (r = 0.60), valine (r = 0.41), methionine (r = 0.62), tryptophan (r = 0.72), and leucine (r = 0.52). There were significant negative correlations between phenylalanine and arginine (r = −0.40), glycine (r = −0.39), and proline (r = −0.54). The classification model based on PCA showed that the first two principal components (PCs) accounted for 51.2% of the total variance, with PC1 and PC2 contributing 31.2% and 20.0% of the total variance, respectively (Figure 1B). Leucine, methionine, glutamine, tryptophan, and methionine were the main factors contributing to PC1. Glycine, total acids, isoleucine, asparagine, and glutamate were the main representative factors of PC2. The 30 eggplant varieties were classified into four categories based on PC1 and PC2: the first category included eight varieties located in the first quadrant (V1, V5, V6, V7, V8, V17, V24, and V30); the second category included nine varieties in the second quadrant (V3, V9, V11, V19, V23, V25, V26, V28, and V29); the third category included eight varieties in the third quadrant (V2, V4, V10, V12, V20, V21, V22, and V27), and; the fourth category included five varieties in the fourth quadrant (V13, V14, V15, V16, and V18).

### 3.2. Difference Analysis of Polyphenol and Anthocyanin Components

#### 3.2.1. Variance and Diversity Analyses of Polyphenol and Anthocyanin Components

There were significant differences in the compositions and contents of polyphenols and anthocyanins among the different eggplant varieties (Table S3). Five green eggplant varieties (V2, V4, V5, V6, V8, V12, and V21) contained significantly lower delphinidin-3-O-rutinoside chloride and delphinidin chloride concentrations than other varieties. The V16, V19, and V24 purple eggplant varieties contained significantly higher delphinidin-3-O-rutinoside chloride concentrations than other varieties, which were 1322-, 1203-, and 1077-times higher, respectively, than that of V8. The V9, V28, and V30 purple eggplant varieties contained significantly higher delphinidin chloride concentrations than other varieties, which were 94-, 90-, and 80-times higher, respectively, than that of V8. Diversity analysis demonstrated that the top three polyphenols among the 30 eggplant varieties were chlorogenic, gentianic, and benzoic acids (Table 2). The standard deviation of polyphenols of different eggplant varieties ranged from 18.45 to 4742.91, and the CV ranged from 21.25 to 74.53%. The standard deviation of anthocyanins of different eggplant varieties ranged from 133.02 to 245.39 and the CV ranged from 78.17 to 102.89%.

#### 3.2.2. Correlation Analysis and PCA of Polyphenol and Anthocyanin Components

**Figure 2:**
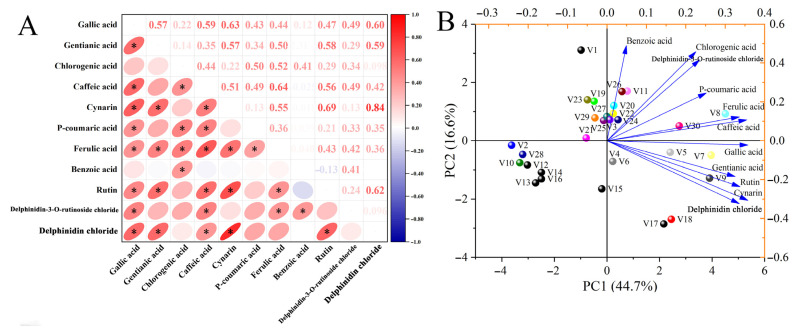
(**A**) Pearson’s correlation analysis and (**B**) principal component analysis of polyphenol and anthocyanin components in different eggplant varieties. Data are mean values (*n* = 3). * indicates a significant correlation (*p* < 0.05) (two-tailed). V1–V30 represent 30 different Chinese eggplant varieties.

Pearson’s correlation analysis was performed to determine the detailed relationship between different polyphenol and anthocyanin parameters (Figure 2A). The results revealed significant positive correlations between among gallic acid and gentianic acid (r = 0.57), caffeic acid (r = 0.59), cynarin (r = 0.63), *p*-coumaric acid (r = 0.43), ferulic acid (r = 0.44), delphinidin-3-O-rutinoside chloride (r = 0.49), and delphinidin chloride (r = 0.60). Chlorogenic acid was significantly correlated with caffeic acid (r = 0.44), *p*-coumaric acid (r = 0.50), ferulic acid (r = 0.52), and benzoic acid (r = 0.41). Rutin was significantly correlated with gallic acid (r = 0.47), gentianic acid (r = 0.58), caffeic acid (r = 0.56), cynarine (r = 0.69), ferulic acid (r = 0.43), and delphinidin chloride (r = 0.62). The classification model for the polyphenol and anthocyanin content of different eggplant varieties was obtained using PCA (Figure 2B). This analysis included 30 varieties and 11 polyphenol and anthocyanin parameters. The first two PCs explained 61.3% of the total variation, with PC1 and PC2 accounting for 44.7% and 16.6%, respectively. The major factors affecting PC1 were gallic acid, caffeic acid, and rutin contents. Chlorogenic acid, benzoic acid, and delphinidin-3-o-rutinoside chloride mainly contributed to PC2. The 30 eggplant varieties were classified into two categories based on PC2: the first category included V1, V3, V8, V11, V19, V20, V21, V22, V23, V24, V25, V26, V27, V29, and V30; the second category included V2, V4, V5, V6, V7, V9, V10, V12, V13, V14, V15, V16, V17, V18, and V28.

### 3.3. Difference Analysis of Total Polyphenol, Total Anthocyanin, VC, and Antioxidant Capacity

#### 3.3.1. Variance Analysis of Total Polyphenol, Total Anthocyanin, VC, and Antioxidant Capacity

**Figure 3:**
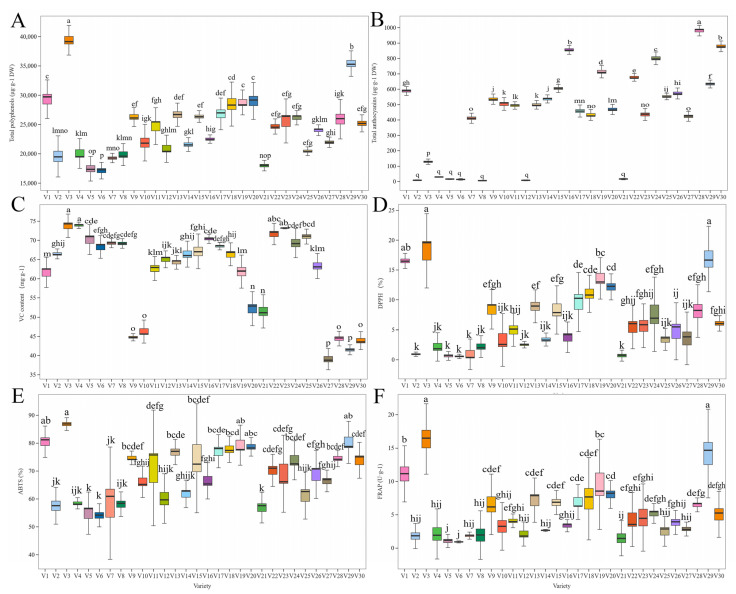
Difference analysis of (**A**) total polyphenols, (**B**) total anthocyanins, (**C**) vitamin C (VC), (**D**) 2,2-diphenyl-1-picrylhydrazyl (DPPH), (**E**) 2,2’-azino-bis(3-ethylbenzothiazoline-6-sulfonic acid) (ABTS), and (**F**) ferric-reducing antioxidant power (FRAP) in 30 eggplant varieties. Different lowercase letters represent significant differences (*p* < 0.05). V1–V30 represent 30 different Chinese eggplant varieties.

As shown in Figure 3, the total polyphenol content was significantly higher in V3 and V29 than in the other 28 varieties. The total polyphenol content of V1 was significantly higher than that of the other 25 varieties, but it was not significantly different from that of V19 and V20. The total anthocyanin content of V28, V30, and V16 was significantly higher than that of the other 27 varieties. Of the purple eggplant varieties, V3 had a significantly lower anthocyanin content than the other 22 varieties. Green eggplant varieties (V2, V4, V5, V6, V8, V12, and V21) had significantly lower total anthocyanin contents than purple varieties. A significantly higher VC content was found in V3, V4, and V23 than in the other eggplant varieties; however, there was no significant difference between these three varieties. V27, V29, and V30 varieties had lower VC content. The DPPH, ABTS, and FRAP antioxidant capacities peaked in V3 (Table S4). In addition to V1 and V29, V3 had a significantly higher DPPH antioxidant capacity than the other 27 varieties. The ABTS antioxidant capacity of V3 was not significantly different from that of V29, V1, V19, and V20, but it was significantly higher than that of the other 26 varieties. The FRAP antioxidant capacities of V3 and V29 were significantly higher than those of the other 28 varieties, followed by that of V1.

#### 3.3.2. Correlation Analysis and PCA of Total Polyphenol, Total Anthocyanin, VC, and Antioxidant Capacity

**Figure 4:**
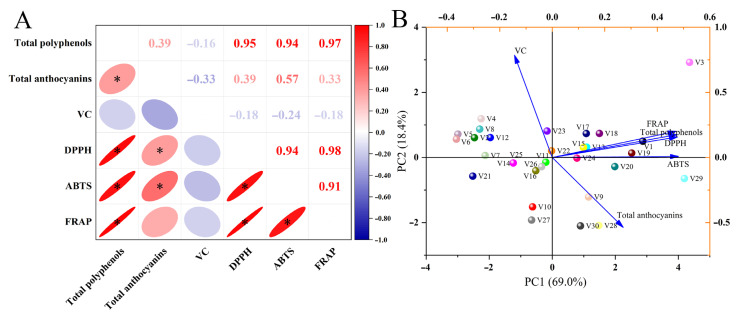
(**A**) Pearson’s correlation analysis and (**B**) principal component analysis of antioxidants and antioxidant capacity in different eggplant varieties. Data are mean values (*n* = 3). * represents a significant correlation (*p* < 0.05) (two-tailed). V1–V30 represent 30 different Chinese eggplant varieties.

There were multiple significant positive correlations between the antioxidant and antioxidant capacity parameters (Figure 4). A significant positive correlation was observed between total polyphenols and anthocyanins (r = 0.39) as well as between total polyphenol content and antioxidant capacity parameters (DPPH, ABTS, and FRAP), with correlation coefficients of 0.95, 0.94, and 0.97, respectively. DPPH was significantly and positively correlated with ABTS (r = 0.94) and FRAP (r = 0.98), and ABTS was significantly and positively correlated with FRAP (r = 0.91). There were significant positive correlations between total anthocyanins and DPPH (r = 0.39) and ABTS (r = 0.57). Furthermore, antioxidants and antioxidant capacity of all eggplant varieties were analyzed by PCA. PC1 and PC2 represented 87.4% of the original data, with PC1 (69.0%) reflecting more than 50% of the information. PC1 mainly synthesized information on FRAP, total polyphenols, DPPH, ABTS, and total anthocyanins. Consequently, two categorizations were derived based on PC1: one for V3, V29, V30, V28, V20, V9, V24, V18, V15, V17, V13, V19, and V1 and another for V27, V10, V16, V23, V22, V26, V11, V25, V14, V7, V21, V6, V54, V12, V8, and V2. Classification 1 means high FRAP, total polyphenols, DPPH, ABTS, and total anthocyanins, with high antioxidant capacity. Classification 2 means lower FRAP, total polyphenols, DPPH, ABTS, and total anthocyanins, with low antioxidant capacity.

### 3.4. Comprehensive Evaluation of Amino Acids, Antioxidants, and Antioxidant Capacity of 30 Chinese Eggplant Varieties Based on PCA

**Figure 5:**
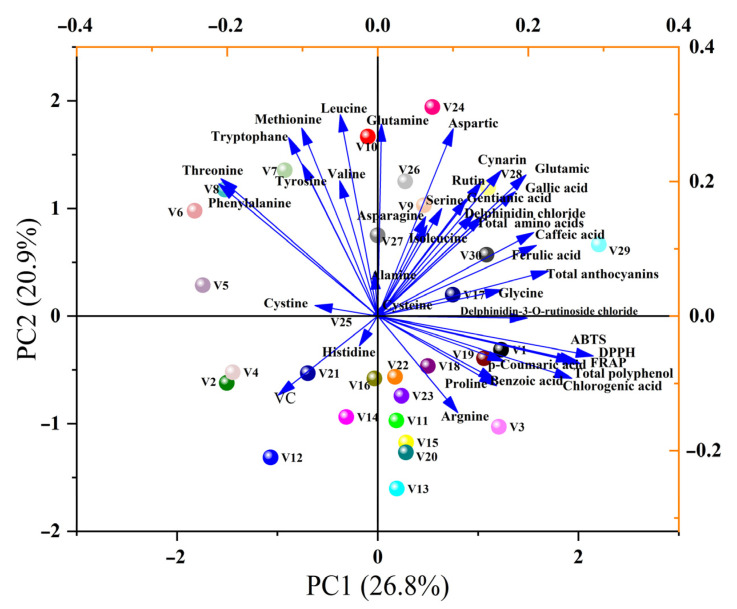
Principal component analysis of amino acids, polyphenols, anthocyanins, VC and antioxidant capacity of different eggplant varieties. V1–V30 represent 30 different Chinese eggplant varieties.

We performed a comprehensive evaluation of 38 indicators in 30 Chinese eggplant varieties using PCA to better compare the differences in fruit quality and antioxidant capacity among different Chinese eggplants. The PCA results extracted eight principal components based on eigenvalues more than 1, with a cumulative variance contribution of 83.34% (Table 3). It showed that the eight principal components responded to 83.34% of the information from the original 38 indicators. The first three principal components reflected more than 50% of the information from the original metrics. Of these, the principal component 1 had the highest variance contribution of 26.82% according to the principal component loading values in Table S5. It mainly incorporated information on antioxidant substances and antioxidant capacity, including ABTS, total polyphenol, DPPH, FRAP, chlorogenic acid, total anthocyanin, caffeic acid, delphinidin-3-O-rutinoside chloride, and ferulic acid. The principal component 2 contributed 20.87% of the variance and mainly combined with information from most of the amino acids including leucine, glutamine, methionine, histidine, tryptophane, tyrosine, valine, and phenylalanine. The variance contribution of principal component 3 was 10.78%, primarily combining information from isoleucine, asparagine, and serine. As can be seen from Figure 5 generated from the first two principal components, the eggplant varieties in the first quadrant (V29, 24, 28, 26, 9, 30, and 17) had higher levels of total amino acids, total polyphenols, total anthocyanins, ABTS, DPPH, and FRAP. Overall, we found the top five highest-scoring varieties in Table 4 were V29, V24, V1, V3, and V28, indicating that they had higher amino acids, antioxidants (polyphenols, anthocyanins, and VC), and antioxidant capacity (DPPH, ABTS, and FRAP).

## 4. Discussion

The nutrient composition and content of eggplants as well as the taste quality of fruits directly affect the commercial value of eggplants. Minimal information is available on the primary metabolite composition of Chinese eggplant varieties despite China being the largest eggplant producer and consumer in the world. Zhou et al. [27] conducted a comprehensive analysis of the metabolomes and transcriptomes of five eggplant varieties with different fruit colors to explore overall information on the flavonoid metabolites of eggplant and to enhance new insights into the regulatory network of fruit coloration. Sugars are vital primary metabolites that have nutritional value and contribute to the sweetness of fruits [28]. Glucose and fructose make up the majority of soluble sugars in most fruits [29,30]. The major soluble sugars in Turkish and Japanese eggplant fruits are fructose, glucose, and sucrose, whereas the major sugars in Chinese eggplant are mannose, glucose, and fructose [2]. Here, we focused on the variation in the primary amino acid metabolite composition among different eggplant varieties. To explore the diversity of primary metabolites in eggplant fruits, we used LC-MS to comprehensively profile amino acid components in biological samples under the same environmental conditions and harvest periods. The LC-MS method has been successfully used to determine the metabolite profiles of a variety of plants, such as tomato [31], date [32], and nigella species seeds [33]. Our findings revealed that the most abundant amino acids in 30 Chinese eggplant varieties were glutamic, arginine, and aspartic; glutamine, asparagine, and lysine were the three most abundant amino acids in Turkish eggplants [3]; the main amino acids found in Japanese eggplants were asparagine, glutamine, and arginine acid [34]. The most enriched amino acids detected in another study on Chinese eggplants were phenylalanine, histidine, and glutamic acid [2]. Variations in the amino acid detection in our study of Chinese eggplant varieties and other eggplant varieties may be attributed to different genetic backgrounds and different metabolite detection techniques. The top five varieties with the highest total free amino acid content were V21, V29, V1, V17, and V26, and the top five varieties with the lowest total free amino acid content were V20, V5, V4, V2, and V14. These results provide valuable information for consumers’ preferential choices of foods with high amino acid levels. For example, some specific amino acids are highly valued as dietary supplements to improve endurance and recovery in athletes [35].

Phenylalanine and tyrosine are catabolically metabolized to the anabolic precursors of the phenylpropanoid pathway in plants. Phenylalanine is converted into cinnamic acid by phenylalanine ammonia lyase (PAL). Expression of PAL-encoding genes is highly regulated by different biotic and abiotic stresses and by conditions that increase the lignin requirement of cell wall components [36]. Cinnamic acid is catalytically metabolized to p-coumaric acid by cinnamic acid 4-hydroxylase. P-coumaric acid may be further metabolized to p-coumaroyl-CoA, a central metabolite of the phenylpropanoid pathway, which is involved in mediating biotic and abiotic stress responses [37]. These compounds confer mechanical strength to plant cells and are involved in plant defenses such as pest deterrence and drought resistance [38,39]. In the present study, the three varieties with the lowest phenylalanine content were V30 (147.63 mg kg^−1^ DW), V23 (173.44 mg kg^−1^ DW), and V13 (196.59 mg kg^−1^ DW). The top three varieties richest in p-coumarin were V13 (275.15 mg kg^−1^ DW), V30 (251.32 mg kg^−1^ DW), and V29 (235.81 mg kg^−1^ DW). The transformation relationships based on the metabolites of the phenylpropanoid metabolic pathway indicated the improved accuracy of the metabolic profiles determined in our investigation.

Phenolic acids, flavonoids, anthocyanins, phenylpropenes, stilbenes, and alkaloids are compounds derived from the phenylpropanoid metabolic pathway, which is involved in plant antioxidant defense and results in plant antioxidant capacity. Furthermore, some of these compounds are involved in the presentation of hues in flowers and fruits [40]. The highly accumulated anthocyanin compounds in eggplant skin, mainly in the presence of glycosidic conjugates of delphinidin, are responsible for a range of black, lilac, and purple hues that characterize the various fruit of eggplant types after domestication [41]. Dephinidin-3-O-rutinoside chloride is a major anthocyanin found in eggplant that scavenges superoxide anions and inhibits lipid peroxidation [42]. Our study revealed that five green eggplant varieties (V2, V4, V5, V6, V8, V12, and V21) contained significantly lower delphinidin-3-O-rutinoside chloride and delphinidin chloride concentrations than other purple eggplant varieties. The DPPH, ABTS, and FRAP values used to describe the antioxidant capacity of the five green varieties were significantly lower than those of the purple varieties. The V16, V19, and V24 purple varieties contained significantly higher delphinidin-3-O-rutinoside chloride concentrations than other varieties. The V9, V28, and V30 purple varieties contained significantly higher delphinidin chloride concentrations than other varieties. The main phenolic acid compound is 5-caffeoylquinic acid, also known as chlorogenic acid, which is considered the main contributor to the antioxidant capacity of eggplant [43]. Among the different phenolic acid types in eggplants, chlorogenic acid is the most common, which accounts for 75–94% of the total phenolic content in various eggplant cultivars [2]. Similarly, in the present study, we found that the most abundant phenolic acid in 30 eggplant varieties was chlorogenic acid (22,319.32 µg g^−1^ DW), which accounted for 91.23% of the total phenolic acids. V3, V29, and V1 were the richest in chlorogenic acid and total polyphenols, and the antioxidant capacities of these three varieties in terms of DPPH, ABTS, and FRAP were also strong. Screening of cultivars and wild varieties with widely varying phenolic acid content is valuable for breeding modern eggplant varieties that are abundant in polyphenols [43]. Furthermore, V3 had a high VC content. VC (ascorbic acid) is well known for its powerful antioxidant activity, reactive oxygen species scavenging ability, and enhanced plant stress resistance [44]. VC is a vital micronutrient and essential antioxidant in the human diet [45] and is present in plants in two water-soluble bioactive forms: ascorbic acid (reduced form) and dehydroascorbic acid (oxidized form). Once synthesized, ascorbic acid (AsA) can be rapidly oxidized owing to its antioxidant function; thus, the recycling pathway (reduction in oxidized forms) also plays a significant role in maintaining AsA levels and the redox state of plant cells [46]. V3 had a strong free-radical scavenging ability because of its high VC and total polyphenol contents.

CV, the main indicator of genetic differences among germplasms, represents the diversity of the traits involved. In general, the CVs of traits with rich genetic backgrounds are large, which is a useful indicator of germplasm identification and evaluation [47]. CV has been used to describe genetic variations in morphological characteristics and nutritional qualities of numerous plants, including pepper [5], peaches [48], and bananas [49]. Additionally, CV < 10%, 10 < CV < 20%, and CV > 20% were defined as indicators of low, medium, and high variation levels, respectively [5]. In the present study, cysteine had a low degree of variability; isoleucine, asparagine, valine, serine, and cystine had a medium degree of variability; the remaining 15 amino acid components were highly variable. For polyphenols and anthocyanins, all indicators showed high variance. In particular, the CV for delphinidin chloride was ≤102.89%. These variations were due to genetic differences, as these eggplant varieties were managed under the same cultivation conditions and daily measures. These results indicated that the amino acids and polyphenols of the 30 eggplant varieties were highly variable, providing useful information for eggplant breeding.

Multivariate statistical analyses included correlation analysis, PCA, and HCA, which were used to visualize correlations, similarities, and differences in multivariate data. Pearson’s correlation analysis revealed multiple sets of significant positive and negative correlations between amino acid components, polyphenols, and anthocyanins in the 30 eggplant varieties. The results revealed an intrinsic relationship between these traits, leading to overlapping information, and they revealed the necessity for further analyses using PCA. PCA is a reliable statistical approach for converting multiple indicators into integrated indicators and has become one of the main methods for comprehensive quality assessment of fruits, vegetables, and other foods [4]. PCA of the amino acid, polyphenol, and anthocyanin fractions of the 30 varieties revealed that both of the extracted PCs could cover >50% of the total information of the original data, implying that the data analysis was reliable. Leucine, methionine, glutamine, tryptophan, methionine, and threonine were the five main amino acids contributing to PC1. Glycine, total acids, isoleucine, asparagine, and glutamic acid were the main factors represented by PC2. Regarding polyphenols and anthocyanins, the major factors representing PC1 were gallic acid, caffeic acid, and rutin. Chlorogenic acid, benzoic acid, and delphinidin-3-o-rutinoside chloride mainly contributed to PC2. The 30 varieties were categorized based on PC1 and PC2, and the classification results provided a reference point for consumer preference choices for certain amino acids, polyphenols, and anthocyanins [50].

Because free radical accumulation results in tissue damage and organ aging, the free radical scavenging capacity (DPPH, ABTS, and FRAP) reflects the antioxidant capacity of the fruit to some extent, which is regarded as an essential tool for measuring fruit and vegetable aging [4,31]. In general, the higher the free radical scavenging rate, the lower the degree of aging and the higher the fruit quality. The antioxidant capacity parameters DPPH, ABTS, and FRAP were highest in V3, followed by V29 and V1. Pearson’s correlation analysis showed a significant positive correlation between total polyphenol content and antioxidant capacity parameters (DPPH, ABTS, and FRAP), with a high correlation coefficient for all parameters. Significant positive correlations were also found between the total anthocyanin content and DPPH and ABTS. These results indicated that polyphenols and anthocyanins contribute significantly to the antioxidant capacity of eggplant, as reflected in several previous studies [40,50]. The HCA-based classification model classified 30 eggplant varieties into three categories. The first category, with high antioxidant capacity, was V3. The second category, with low antioxidant capacity, included V27, V10, V16, V23, V22, V26, V11, V25, V14, V7, V21, V6, V54, V12, V8, and V2. The third category, with medium antioxidant capacity, included V29, V30, V28, V20, V9, V24, V18, V15, V17, V13, V19, and V1.

Our previously mentioned study used PCA or HCA to individually classify the amino acids, antioxidants, and antioxidant capacity of 30 Chinese eggplant varieties to meet some breeder/consumer preferences for one of these categories. Meanwhile, it is desired, for consumers and researchers, to screen eggplant varieties for both of these properties. It was noted that PCA can be used not only for intuitive visual categorization but also for comprehensive ranking [31,51]. Shi et al. [52] used PCA to rank the metabolic characteristics and quality of 20 jujube varieties from seven main production areas in China. It is visualized in Figure 5 that the eggplant varieties in the first quadrant (V29, V24, V28, V26, V9, V30, and V17) had higher levels of total amino acids, total polyphenols, total anthocyanins, ABTS, DPPH, and FRAP. Hence, we further ranked different Chinese eggplant varieties using PCA and obtained the top five eggplants possessing higher amino acids, antioxidants, and antioxidant capacity as V29, V24, V1, V3, and V28, respectively.

## 5. Conclusions

The present study revealed high variability in amino acid, polyphenol, and anthocyanin traits, particularly histidine and delphinidin chloride contents, by analyzing the nutritional quality of 30 eggplant varieties as the main cultivars distributed throughout China. V21 (‘Wanqie 048’; 26,827.40 mg kg^−1^ DW) had the highest total amino acid content. Total polyphenol content was highest in V3 (‘Zhengqie924’; 39,474.98 µg g^−1^ DW), followed by V29 and V1. The highest total anthocyanin content was in V28 (‘E150725’; 978.32 µg g^−1^ DW). The total anthocyanin content of the seven green eggplant varieties (V2, V4, V5, V6, V8, V12, and V21) was lower than those of the purple varieties. Higher antioxidant capacities were observed in V3 (‘Zhengqie924’), V29 (‘Zhengqie 903’), and V1 (‘1871’). V3 (‘Zhengqie924’), V4 (‘1942’), and V23 (‘Rong 17-66’) are eggplant varieties with higher VC content. We processed the amino acid, polyphenol, and anthocyanin data using multivariate statistical analyses for improved visualization and reliability of the results. Pearson’s correlation analysis revealed a significant contribution of polyphenols and anthocyanins to the antioxidant capacity of eggplant. The PCA-based classification model for amino acids, polyphenols, and anthocyanins could provide guidelines for consumer preference selection. A classification model based on PCA classified the 30 Chinese eggplant varieties into three categories: high, medium, and low antioxidant capacities. PCA-based ranking revealed that the top five Chinese eggplant varieties with higher amino acids, antioxidants, and antioxidant capacity were V29 (‘Zhengqie903’), V24 (‘Zhengqie78’), V1 (‘1871’), V3 (‘Zhengqie924’), and V28 (‘E150725’). This study comprehensively evaluated the nutritional traits of eggplant varieties, providing valuable data for parental selection in subsequent breeding programs to optimize eggplant varieties with high nutritional qualities to more effectively meet market and consumer demands.

## Figures and Tables

**Table 1:** Estimates of descriptive statistics (including the min., max., mean, SD, and CV) for free amino acids (mg kg^−1^ DW) contents of fruit of 30 Chinese eggplant varieties.

Amino Acid	Min.	Max.	Mean	SD	CV (%)
Threonine	176.29	879.38	383.42	185.98	48.50
Phenylalanine	147.63	740.55	323.13	154.71	47.88
Leucine	59.68	195.22	111.35	34.25	30.76
Isoleucine	370.75	958.76	571.09	109.9	19.24
Asparagine	360.29	944.14	583.62	110.22	18.89
Tryptophane	54.78	482.66	165.63	122.10	73.72
Methionine	23.14	71.85	42.40	16.00	37.75
Proline	387.40	2079.18	1066.55	454.49	42.61
Valine	397.59	987.18	634.48	117.97	18.59
Tyrosine	25.21	150.90	54.32	27.81	51.21
Cysteine	12.07	15.08	12.95	0.76	5.85
Alanine	570.14	1095.32	883.48	121.00	13.70
Glycine	12.23	129.60	77.64	27.06	34.85
Serine	490.67	802.41	627.62	88.59	14.12
Glutamate	4083.11	8343.35	5978.31	1263.78	21.14
Aspartate	1833.99	4088.20	2869.42	645.91	22.51
Histidine	340.71	9253.85	1774.78	1883.77	106.14
Cystine	6.83	12.19	9.67	1.61	16.62
Argnine	406.94	5689.72	2912.62	1265.72	43.46
Glutamine	240.35	1818.28	924.20	347.15	37.56
Total amino acids	15,267.19	26,827.40	20,006.69	2899.02	14.49

Note: Note: SD = standard deviation; CV = coefficient of variation; DW = dry weight.

**Table 2:** Estimates of descriptive statistics (including the min., max., mean, SD, and CV) for the polyphenol and anthocyanin (μg·g^−1^ DW) contents in fruit of 30 eggplant varieties.

Index		Min.	Max.	Mean	SD	CV (%)
Polyphenol	Gallic acid	19.57	93.02	55.98	18.45	32.95
	Gentianic acid	439.34	1462.09	822.25	251.29	30.56
	Chlorogenic acid	15,483.44	37,105.87	22,319.32	4742.91	21.25
	Caffeic acid	91.50	267.22	171.08	52.64	30.77
	*p*-Coumaric acid	100.41	275.15	169.31	51.46	30.39
	Ferulic acid	31.68	865.31	223.33	166.44	74.53
	Benzoic acid	233.74	1043.21	478.04	145.92	30.52
	Rutin	16.45	148.78	59.05	36.58	61.94
	Cynarin	68.50	423.58	166.11	99.59	59.95
Anthocyanin	Delphinidin-3-O-rutinoside chloride	0.63	832.89	313.91	245.39	78.17
	Delphinidin chloride	3.75	439.87	129.28	133.02	102.89

Note: Note: SD = standard deviation; CV = coefficient of variation; DW = dry weight.

**Table 3:** Eigenvalues and variance contributions of amino acids, antioxidants, and antioxidant capacity of fruits of different Chinese eggplant varieties.

Component Number	Eigenvalues	% of Variance	Cumulative %
1	10.19	26.82	26.82
2	7.93	20.87	47.69
3	4.10	10.78	58.47
4	2.38	6.25	64.72
5	2.24	5.90	70.63
6	1.91	5.04	75.66
7	1.72	4.53	80.20
8	1.19	3.14	83.34

**Table 4:** Comprehensive scores of amino acids, antioxidants, and antioxidant capacity of fruits of different Chinese eggplant varieties.

Varieties	Score of PC1	Score of PC2	Score of PC3	Score of PC4	Score of PC5	Score of PC6	Score of PC7	Score of PC8	Total Score	Ranking
V1	1.23	−0.31	3.49	0.28	−1.14	0.64	−1.52	1.48	0.60	3
V2	−1.50	−0.62	0.51	−0.70	0.87	−0.20	0.39	−1.80	−0.52	29
V3	1.21	−1.03	0.58	1.22	2.44	1.39	0.74	0.06	0.50	4
V4	−1.45	−0.52	0.15	0.13	0.11	1.32	−0.30	−1.34	−0.45	27
V5	−1.74	0.29	0.09	0.45	0.70	−0.26	0.09	0.17	−0.33	24
V6	−1.82	0.98	0.30	0.68	0.26	0.20	0.75	1.23	−0.11	16
V7	−0.93	1.36	0.03	0.88	−1.05	0.72	−0.08	0.91	0.09	12
V8	−1.52	1.18	0.69	0.22	1.05	0.62	0.78	0.84	0.08	14
V9	0.46	1.03	−0.29	−1.15	1.21	1.08	−0.43	−0.74	0.32	7
V10	−0.10	1.67	−0.51	−1.30	−0.07	1.28	−0.99	−0.37	0.19	10
V11	0.19	−0.97	0.02	0.62	−0.93	−0.52	0.12	0.81	−0.16	17
V12	−1.07	−1.31	0.28	−0.18	−0.53	0.82	−0.07	−0.53	−0.55	30
V13	0.19	−1.60	−1.46	−0.08	1.39	−0.53	−0.26	1.44	−0.36	25
V14	−0.31	−0.94	−0.90	0.45	−1.19	−0.58	0.09	−1.15	−0.48	28
V15	0.28	−1.17	−0.85	−0.80	0.86	−0.48	0.26	1.77	−0.22	20
V16	−0.03	−0.58	0.30	−0.03	0.08	−2.08	−0.96	−0.27	−0.25	22
V17	0.75	0.20	0.94	−0.01	−1.29	0.63	1.94	−1.35	0.34	6
V18	0.50	−0.46	0.21	0.52	−0.47	1.36	−0.18	0.76	0.15	11
V19	1.06	−0.39	0.99	0.56	0.25	−0.41	−0.83	−1.35	0.26	9
V20	0.28	−1.27	−0.96	−0.02	0.87	1.19	−1.47	−0.06	−0.25	21
V21	−0.70	−0.53	1.12	−3.27	−0.45	−1.49	1.37	1.03	−0.39	26
V22	0.17	−0.56	0.11	0.57	−0.96	−0.99	−0.74	−0.08	−0.17	18
V23	0.24	−0.74	−0.74	0.88	−1.21	−0.15	1.40	−1.35	−0.17	19
V24	0.54	1.94	0.13	1.85	1.31	−2.19	−0.32	−0.59	0.61	2
V25	−0.58	−0.05	−1.28	1.34	−1.07	−0.62	0.24	0.66	−0.28	23
V26	0.27	1.25	−0.61	0.22	−0.80	0.01	0.24	0.70	0.27	8
V27	0.00	0.75	0.44	−1.27	0.51	−1.20	−1.78	−1.27	−0.03	15
V28	1.10	1.19	−1.09	−0.31	−0.25	−0.33	−0.67	0.67	0.37	5
V29	2.20	0.66	0.14	−0.46	0.82	−0.26	2.70	−0.17	0.87	1
V30	1.08	0.57	−1.84	−1.29	−1.31	1.05	−0.51	−0.12	0.08	13

## Data Availability

Data are contained in the article.

## Supplementary Materials

The following supporting information can be downloaded at https://www.mdpi.com/article/10.3390/antiox14010010/s1. Table S1: Information on 30 eggplant varieties; Table S2: Contents of amino acid components (mg kg^−1^ DW) in 30 eggplant varieties; Table S3: Contents of polyphenol and anthocyanin components (μg·g^−1^DW) in 30 eggplant varieties; Table S4: Difference analysis of total polyphenols, total anthocyanins, vitamin C (VC), 2,2-diphenyl-1-picrylhydrazyl (DPPH), 2,2′-azino-bis(3-ethylbenzothiazoline-6-sulfonic acid) (ABTS), and ferric-reducing antioxidant power (FRAP) in 30 eggplant varieties; Table S5: Compositional matrix of amino acids, antioxidants, and antioxidant capacity of different Chinese eggplant varieties.
