# Supplementary material for: A Comprehensive Evaluation of Nutritional Quality and Antioxidant Capacity of Different Chinese Eggplant Varieties Based on Multivariate Statistical Analysis
Source: Antioxidants (Basel). 2024 Dec 25;14(1):10. doi: 10.3390/antiox14010010 (PMC11761265; doi:10.3390/antiox14010010)
Supplement: Supplementary file 1 [file antioxidants-14-00010-s001.zip › antioxidants-3351307-supplementary.pdf]

Table S1 Information on 30 eggplant varieties

Number	Variety name	Fruit shape	Peel color	Origin	Availability Methods
V1	1871	Round	Purple	Zhumadian Institute of Agricultural Science	available in the market
V2	Zhengqie907	Round	Green	Zhengzhou Vegetable Research Institute	available in the market
V3	Zhengqie924	Round	Purple	Zhengzhou Vegetable Research Institute	available in the market
V4	1942	Round	Green	Zhumadian Institute of Agricultural Science	available in the market
V5	1908	Round	Green	Zhumadian Institute of Agricultural Science	available in the market
V6	Zhengqie908	Round	Green	Zhengzhou Vegetable Research Institute	available in the market
V7	420	Round	Purple	Institute of Vegetables and Flowers, Chinese Academy of Agricultural Sciences	available in the market
V8	1919	Round	Green	Zhumadian Institute of Agricultural Science	available in the market
V9	cw213	Round	Purple	Institute of Vegetables and Flowers, Chinese Academy of Agricultural Sciences	available in the market
V10	1777	Round	Purple	Zhumadian Institute of Agricultural Science	available in the market
V11	216	Long	Purple	Institute of Vegetables and Flowers, Chinese Academy of Agricultural Sciences	available in the market
V12	Lvtianshi	Long	Green	Shanghai Academy of Agricultural Sciences	available in the market
V13	Yuzaoqie9	Long	Purple	Chongqing Academy of Agricultural Sciences	available in the market
V14	E160516	Long	Purple	Jilin Scientific Research Institute of Vegetables and Flowers	available in the market
V15	19-824	Long	Purple	Chongqing Academy of Agricultural Sciences	available in the market
V16	Weichangqie101	Long	Purple	Weifang Academy of Agricultural Sciences, Shandong Province	available in the market
V17	Yuqie 6	Long	Purple	Chongqing Academy of Agricultural Sciences	available in the market
V18	Rong 19-14	Long	Purple	Chengdu Academy of Agriculture and Forestry Sciences	available in the market
V19	Huhei 6	Long	Purple	Shanghai Academy of Agricultural Sciences	available in the market
V20	Changqie 719	Long	Purple	Vegetable Research Institute of Shandong Academy of Agricultural Sciences	available in the market
V21	Wanqie 048	Long	Green	Horticulture Institute of Anhui Academy of Agricultural Sciences	available in the market
V22	Weichangqie 71	Long	Purple	Weifang Academy of Agricultural Sciences, Shandong Province	available in the market
V23	Rong 17-66	Long	Purple	Chengdu Academy of Agriculture and Forestry Sciences	available in the market
V24	Weichangqie 78	Long	Purple	Weifang Academy of Agricultural Sciences, Shandong Province	available in the market
V25	Huqie 316	Long	Purple	Shanghai Academy of Agricultural Sciences	available in the market
V26	Changqie 1016	Long	Purple	Vegetable Research Institute of Shandong Academy of Agricultural Sciences	available in the market
V27	Zhengqie 809	Long	Purple	Zhengzhou Vegetable Research Institute	available in the market
V28	E150725	Long	Purple	Jilin Scientific Research Institute of Vegetables and Flowers	available in the market
V29	Zhengqie 903	Long	Purple	Zhengzhou Vegetable Research Institute	available in the market
V30	1952	Long	Purple	Zhumadian Institute of Agricultural Science	available in the market

Table S2 Contents of amino acid components (mg kg^{-1} DW) in 30 eggplant varieties

Varieties	Threonine	Phenylalanine	Leucine	Isoleucine	Asparagine	Tryptophane	Methionine	Proline	Valine	Tyrosine
V1	273.99±9.83 mn	238.85±9.60 lm	106.91±1.46 hi	958.76±9.38 a	944.14±9.63 a	84.76±6.12 jkl	25.88±0.96 lmn	2079.18±38.79 a	605.75±19.03 ijk	33.96±1.38 o
V2	577.98±5.77 f	487.17±6.16 e	91.23±1.46 klm	555.56±8.31 hijk	582.33±9.91 hij	134.62±1.29 i	26.40±0.80 lmn	532.86±27.86 no	720.71±27.38 cdef	50.94±1.44 gh
V3	283.18±3.81 m	244.87±5.54 kl	86.28±1.21 mn	532.44±9.57 jkl	582.42±9.21 hij	54.78±2.80 o	30.07±0.98 ijk	1200.84±16.61 h	514.55±14.67 mn	37.09±1.15 mno
V4	606.43±8.63 de	514.44±9.93 d	87.45±1.15 mn	580.97±9.13 gh	559.42±9.34 jkl	133.95±5.57 i	27.58±0.96 klm	522.05±14.45 no	738.95±18.66 bcde	38.31±1.02 lmno
V5	588.16±3.99 ef	496.24±2.49 de	159.48±1.38 d	524.98±1.10 kl	522.5±9.44 m	283.98±2.77 e	47.69±0.95 f	445.35±15.94 op	680.51±13.93 efgh	110.61±1.16 b
V6	739.31±8.64 b	622.27±9.58 b	154.74±1.60 d	574.41±3.46 gh	600.24±8.94 ghi	482.66±5.8 a	64.82±0.90 c	387.40±15.16 p	779.11±11.69 bcde	92.16±1.25 c
V7	660.85±8.65 c	557.98±9.93 c	179.37±1.92 b	663.84±3.43 cd	692.28±9.66 cd	335.26±8.24 c	71.22±0.93 a	1290.44±29.55 gh	659.10±14.07 fghi	91.21±1.10 c
V8	879.38±5.27 a	740.55±9.44 a	117.29±1.13 g	553.68±3.94 hijk	575.69±9.62 ij	337.23±6.27 c	71.85±0.93 a	465.32±17.39 op	760.15±21.62 bcd	80.28±1.33 d
V9	463.94±9.70 h	384.45±9.06 gh	112.00±1.04 h	574.44±8.06 gh	577.26±3.44 ij	235.50±10.38 f	41.54±0.92 g	801.91±18.33 m	669.58±14.77 fghi	42.90±1.42 jkl
V10	619.66±2.82 d	515.53±5.12 d	134.39±1.22 f	674.82±9.93 c	718.74±9.22 bc	148.26±2.63 hi	69.48±0.92 ab	604.30±15.22 n	795.09±17.86 b	52.75±1.46 gh
V11	257.38±5.09 no	217.16±5.43 mn	89.67±1.39 lm	532.17±5.14 jkl	558.69±7.20 jkl	76.99±1.23 klm	28.76±0.71 jkl	1633.98±19.45 cd	556.66±13.76 klmn	36.62±1.05 mno
V12	368.26±3.04 j	313.02±1.11 i	82.46±1.25 no	534.67±4.15 jkl	529.00±7.81 lm	55.18±1.11 o	24.79±0.35 mn	961.42±17.29 jk	506.99±13.95 n	38.06±1.22 lmno
V13	230.53±6.34 op	196.59±5.67 no	60.33±1.53 q	370.75±6.87 o	360.29±8.45 o	62.33±1.70 mno	23.14±0.68 o	923.75±19.02 kl	397.59±12.84 o	34.71±1.03 no
V14	237.77±3.57 op	198.58±2.69 no	80.04±1.44 o	528.70±7.84 kl	539.16±9.94 klm	95.50±4.51 jk	43.09±0.83 g	1585.83±29.76 cde	577.92±12.59 klm	40.39±1.46 jklm
V15	236.80±4.74 op	200.84±5.54 no	59.68±1.17 q	383.36±6.19 no	389.85±6.88 o	64.54±2.17 mno	31.59±0.61 ij	1050.00±15.85 ij	519.89±13.70 lmn	38.23±1.09 lmno
V16	288.20±4.02 lm	248.32±6.31 kl	97.51±1.47 j	553.25±6.83 hijk	572.19±6.73 ijk	77.78±1.55 klm	32.80±0.84 i	461.45±16.2 op	508.69±13.02 mn	42.97±1.03 jkl
V17	249.17±2.91 no	208.54±3.19 no	143.71±1.61 e	672.40±7.00 c	656.46±7.54 ef	76.14±1.73 klmn	30.49±0.52 ijk	1554.39±15.98 def	987.18±19.03 a	35.32±1.29 mno
V18	248.21±4.39 no	211.75±3.97 no	93.9 ±1.20 jkl	581.01±3.98 gh	604.28±5.87 ghi	81.96±1.51 klm	41.68±0.87 g	1648.30±48.57 c	649.50±13.42 ghij	43.51±1.21 jk
V19	250.34±6.89 no	217.77±8.70 mn	89.89±1.36 lm	716.74±9.85 b	734.10±7.38 b	62.65±4.09 mno	26.16±0.53 lmn	1274.44±40.94 gh	586.69±19.11 jkl	39.57±1.15 klmn
V20	232.94±2.70 op	200.63±6.01 no	71.75±1.13 p	453.85±3.65 m	460.28±9.19 n	66.49±1.68 lmno	31.42±0.95 ij	1094.04±14.08 i	551.96±14.32 klmn	33.29±1.56 o
V21	213.92±5.57 pq	191.81±6.20 no	109.66±1.50 hi	571.61±9.35 ghi	589.56±6.83 hij	56.85±1.07 no	47.44±0.72 f	876.79±14.12 klm	606.19±16.10 ijk	25.43±1.03 p
V22	312.53±7.93 kl	270.97±9.19 jk	98.84±1.78 j	561.10±9.28 hij	592.19±9.22 hij	90.79±6.65 jk	32.61±0.71 i	1470.03±39.57 f	491.31±23.44 n	44.96±1.27 ij
V23	192.84±4.92 qr	173.44±9.83 o	98.72±1.28 j	541.70±9.74 ijk	511.39±9.88 m	93.66±7.58 jk	30.32±0.91 ijk	1502.79±18.72 ef	653.11±13.07 fghij	54.57±1.42 g
V24	533.19±7.45 g	435.99±8.32 f	195.22±1.56 a	660.04±9.50 cd	667.51±9.22 de	366.86±2.64 b	61.19±0.90 d	1025.83±14.93 ij	603.78±21.26 ijk	150.90±1.27 a

V25	431.44±9.65 i	358.00±7.45 h	111.56±1.18 h	409.61±7.81 n	444.98±6.33 n	366.39±3.68 b	66.68±0.60 bc	1871.60±11.01 b	550.04±13.47 klmn	75.22±1.36 e
V26	466.59±8.03 h	374.27±9.72 gh	170.30±1.91 c	638.16±7.72 de	631.83±9.13 fg	297.65±8.42 de	66.48±0.8 bc	857.64±12.29 lm	710.55±16.30 defg	55.74±1.11 g
V27	253.03±9.93 no	198.01±1.44 no	119.45±1.74 g	619.66±4.20 ef	665.2±5.85 de	183.46±3.18 g	53.38±0.99 e	802.67±14.95 m	588.48±14.02 jkl	62.78±1.03 f
V28	334.28±4.59 k	278.01±1.41 jk	137.90±1.24 f	504.54±4.34 l	520.91±7.43 m	304.98±6.38 de	46.48±0.82 f	1015.37±19.62ij	676.33±15.09 efgh	73.40±1.13 e
V29	296.08±6.40 lm	250.33±4.55 kl	104.88±1.53 i	596.04±9.76 fg	615.16±9.82 gh	154.82±6.39 h	37.04±0.96 h	1194.65±18.18 h	613.96±14.75 hijk	48.46±1.49 hi
V30	176.29±2.19 r	147.63±1.77 p	96.00±1.32 jk	509.41±8.76 l	510.60±9.10 m	102.80±2.30 j	39.83±0.68 gh	861.91±23.75 lm	774.06±18.34 bcd	25.21±1.34 p

Note: Different lowercase letters in the table represents significant differences at the $p < 0.05$ level.

Continue Table S2 Contents of amino acid components (mg kg^{-1} DW) in 30 eggplant varieties

Varieties	Cysteine	Alanine	Glycine	Serine	Glutamate	Aspartate	Histidine	Cystine	Argnine	Glutamine	Total amino acids
V1	13.15±0.48 bcd	1095.32±23.46 a	129.60±0.90 a	802.41±23.32 a	5728.54±120.30 efg	2921.34±90.06 def	2137.99±98.06 ef	11.69±0.24 abc	4811.70±94.54 bc	1216.83±36.05 c	24220.76±544.64c
V2	15.08±0.58 a	810.29±30.45 ghij	60.04±0.86 p	600.40±24.36 fghi	4083.11±135.01 i	1997.99±95.96 mn	2307.93±99.92 ef	9.87±0.37 defg	2224.12±83.49 jkl	830.94±35.61 ghi	16699.59±393.85ghi
V3	13.18±0.53 bcd	969.94±21.37 cd	80.44±0.90 h	663.11±17.15 cdef	6203.47±139.61 cde	2349.97±85.86 ijkl	648.88±30.47 lm	8.53±0.47 ghij	3309.20±47.66 fgh	733.40±23.92 ijkl	18546.63±397.38ijkl
V4	12.37±0.27 cd	842.87±29.04 fghij	65.65±0.88 lmn	529.41±29.47 ijk	4330.52±112.49 i	2735.05±96.07 efgh	651.70±59.26 lm	11±0.29 abcd	1856.40±71.61 lm	782.33±26.02 hij	15626.85±467.99hij
V5	12.85±0.5 bcd	1016.88±15.07 abc	62.14±0.97 op	633.55±15.78 efgh	4348.96±141.49 i	2424.95±34.28 ijk	1582.70±90.55 hi	10.55±0.59 bcde	2136.07±30.67 jk	761.14±34.02 ijk	16849.3±243.92ijkl
V6	12.82±0.43 bcd	957.18±23.68 cd	12.23±0.58 r	686.12±12.38 cde	5387.75±147.80 gh	2632.73±66.77 fghi	2039.72±99.38 efg	9.80±0.32 defg	2190.61±71.66 jk	1159.54±20.99 cd	19585.64±259.35cd
V7	12.77±0.26 bcd	842.07±12.71 fghij	14.70±0.72 r	661.31±11.46 cdef	6839.51±142.87 bcd	3333.21±36.62 cd	1010.21±88.98 kl	11.73±0.59 abc	2161.76±57.89 jk	1076.69±37.50 de	21165.51±212.02de
V8	12.66±0.17 cd	1080.45±26.27 ab	71.19±0.81 jk	729.35±23.19 abcd	6003.94±143.07 def	3700.59±81.26 b	1406.75±93.92 i	10.40±0.31 bcdef	2076.46±56.66 jklm	1429.36±42.77 b	21102.55±433.66b
V9	12.59±0.33 cd	998.72±19.09 bc	99.48±0.65 e	737.92±20.71 abcd	6471.25±129.22 bcd	3122.18±76.42 cd	2239.90±46.53 e	8.26±0.41 ghij	1187.52±34.96 n	1241.15±38.75 c	20022.48±327.27c
V10	12.29±0.13 cd	822.59±13.50 fghij	87.16±0.96 g	554.78±12.80 hijk	5667.07±103.94 efg	3391.92±73.46 c	3333.11±85.38 d	10.58±0.51 bcde	1988.93±34.15 klm	1818.28±51.28 a	22019.72±294.14a
V11	12.26±0.28 cd	897.85±18.20 defg	62.98±0.90 nop	658.49±25.09 def	5294.70±108.92 gh	1994.38±41.40 mn	340.71±16.84 n	7.26±0.13 ij	5025.09±31.53 bc	797.78±27.01 ghij	19079.55±114.98ghij
V12	12.32±0.29 cd	779.27±11.62 j	70.79±0.88 jk	561.06±21.52 hijk	4844.30±138.39 h	2106.59±90.05 lmn	407.47±25.59 mn	11.92±0.27 abc	4584.52±28.95 c	740.02±19.77 ijkl	17532.11±148.42ijkl
V13	12.40±0.27 cd	962.72±26.19 cd	54.17±0.78 q	507.65±18.62 jk	4152.12±150.08 i	1833.99±28.19 n	4273.56±87.54 c	7.79±0.33 hij	2104.34±41.77 jkl	475.48±12.83 n	17044.23±373.1n
V14	12.50±0.38 cd	662.72±17.22 k	66.83±0.89 lm	490.67±23.73 k	4999.56±123.96 h	2242.56±67.84 jklm	452.65±42.71 mn	8.33±0.42 ghij	3275.70±67.94 fgh	634.75±13.09 lm	16773.25±368.98lm
V15	12.79±0.32 bcd	1004.34±17.91 bc	56.22±0.85 q	513.18±19.79 ijk	5281.18±134.87 gh	2143.56±19.89 klm	6095.05±99.2 b	10.58±0.34 bcde	3366.76±71.78 fg	598.25±16.02 m	22056.66±306.02m
V16	14.16±0.83 abc	1066.32±24.59 ab	82.55±0.91 h	557.11±14.73 hijk	6096.90±139.38 cdef	2468.74±43.71 hij	648.22±67.53 lm	8.91±0.42 efghi	4138.59±97.66 de	894.49±36.26 fgh	18859.15±422.48fgh

V17	12.76±0.30 bcd	799.99±17.61 ij	76.81±0.88 i	703.01±14.94 bcde	8343.35±138.94 a	2949.76±26.26 de	1800.19±41.68 gh	8.86±0.48 efghi	3434.02±29.95 f	832.18±22.27 ghi	23574.73±239.95ghi
V18	12.19±0.45 cd	885.60±22.56 defghi	72.43±0.71 jk	698.01±14.57 bcde	6118.05±131.30 cdef	3112.51±15.47 cd	863.35±76.19 kl	11.8±0.51 abc	3106.22±36.04 h	816.59±28.82 ghij	19900.85±232.83ghij
V19	13.04±0.38 bcd	869.69±25.78 efghi	119.20±0.87 c	686.95±26.46 cde	6489.54±137.96 bcd	2636.36±95.12 fghi	601.81±19.89 lmn	8.93±0.22 efghi	2777.57±98.12 i	969.28±30.54 ef	19170.72±495.57ef
V20	12.74±0.60 bcd	803.77±15.55 hij	68.72±0.53 kl	520.48±19.32 ijk	4270.57±134.10 i	2211.95±95.11 jklm	1869.79±99.31 fg	12.53±0.49 abc	1847.64±25.88 m	452.37±24.71 n	15267.19±272.43n
V21	13.97±0.84 abcd	904.00±21.70 def	122.68±0.93 b	668.61±14.64 cdef	5621.99±147.59 fg	2841.86±98.81 defg	9253.85±95.40 a	8.17±0.37 ghij	3496.30±96.67 f	606.70±24.04 m	26827.4±450.85m
V22	13.10±0.38 bcd	831.14±25.37 fghij	87.62±0.93 g	535.94±24.85 ijk	6143.67±146.08 cdef	2739.86±74.99 efgh	634.24±32.22 lm	9.56±0.21 defgh	4327.35±99.53 d	906.44±36.46 fg	20194.26±482.31fgh
V23	12.46±0.26 cd	570.14±18.79 l	63.11±0.93 nop	591.51±18.57 fghij	5956.77±121.39 def	3362.19±98.61 cd	673.34±44.56 lm	7.67±0.10 6ij	3276.68±59.09 fgh	240.35±33.85 o	18606.79±320.36o
V24	13.39±0.32 abcd	944.22±23.62 cde	118.12±0.92 c	747.72±29.83 abc	7818.33±123.98 a	3848.66±59.81 ab	830.37±34.4 kl	8.21±0.49 ghij	406.94±35.48 o	1375.69±35.06 b	20812.16±248.29b
V25	12.16±0.49 d	690.68±14.56 k	64.88±0.92 mno	571.96±13.27 ghijk	4864.37±141.69 h	2599.45±70.38 ghi	669.01±29.38 lm	8.16±0.59 ghij	2610.66±68.75 i	703.46±17.40 jklm	17480.33±191.44jklm
V26	12.66±0.24 cd	891.30±16.91 defgh	85.88±0.95 g	532.51±15.11 ijk	6600.24±142.71 bc	4049.12±78.11 ab	690.28±32.03 lm	10.29±0.49 cdef	3986.79±61.66 e	1353.47±16.26 b	22481.74±407.46b
V27	14.66±0.71 ab	964.04±18.96 cd	112.71±0.93 d	690.99±20.36 cde	6848.88±126.35 b	2963.41±94.88 de	1568.52±76.54 hi	10.86±0.54 abcd	569.22±68.25 o	1419.95±22.96 b	18709.37±344.22b
V28	13.04±0.27 bcd	898.7±15.29 defg	86.99±0.58 g	652.89±24.95 defg	8192.92±135.13 a	3379.87±84.29 cd	1339.88±95.69 ij	8.75±0.35 fghi	2265.54±29.76 j	1145.92±25.21 cd	21876.72±303.93cd
V29	14.19±0.64 abc	818.6±14.23 fghij	91.32±0.84 f	781.22±15.39 ab	8208.27±137.10 a	3899.63±88.21 ab	1769.06±83.88 gh	6.83±0.50 j	5689.72±76.19 a	1065.43±23.48 de	26255.69±498.12de
V30	12.07±0.12 d	823.05±12.64 fghij	82.60±0.94 h	560.17±19.26 hijk	8139.58±116.88 a	4088.20±57.61 a	1103.13±88.4 jk	12.19±0.37 ab	3146.31±26.15 gh	647.79±24.61 klm	21858.82±349.28klm

Note: Different lowercase letters in the table represents significant differences at the $p < 0.05$ level.

Table S3 Contents of polyphenol and anthocyanin components (μg·g^{-1}DW) in 30 eggplant varieties

Varieties	Polyphenol									Anthocyanin	
	Gallic acid	Gentianic acid	Chlorogenic acid	Caffeic acid	P-coumaric acid	Ferulic acid	Benzoic acid	Rutin	Cynarin	Delphinidin-3-O-rutinoside chloride	Delphinidin chloride
V1	55.11 ± 0.61 i	670.16 ± 74.33 klmno	26909.63 ± 639.51 c	137.95 ± 2.76 ijklm	121.27 ± 5.17 lmnop	128.31 ± 3.73 mno	1043.21 ± 47.79 a	21.23 ± 0.84 s	117.71 ± 3.4 ghi	550.75 ± 6.58 d	36.97 ± 0.25 q
V2	25.86 ± 0.83 o	499.85 ± 21.67 op	18350.48 ± 808.82 jkl	119.63 ± 6.08 klmn	148.65 ± 5.67 j	31.68 ± 6.04 q	311.12 ± 51.4 lmn	24.74 ± 0.92 r	81.42 ± 1.67 lm	2.65 ± 0.67 p	5.6 ± 0.17 u
V3	45.83 ± 0.48 kl	834.04 ± 15.58 ghijk	37105.87 ± 570.6 a	182.25 ± 1.64 fg	211.59 ± 3.29 efg	287.4 ± 8.35 gh	574.96 ± 18.72 bcde	97.95 ± 0.83 e	135.09 ± 4.25 fgh	86.89 ± 3.68 o	42.07 ± 0.66 op
V4	30.42 ± 0.95 n	695.69 ± 51.65 klmn	18684.24 ± 592.47 jk	96.49 ± 2.65 op	100.41 ± 2.49 p	109.11 ± 8.47 no	421.82 ± 15.08 ghijkl	32.88 ± 0.88 nop	81.18 ± 3.14 lm	1.15 ± 0.41 p	27.99 ± 0.82 r
V5	44.27 ± 0.8 kl	741.19 ± 59.57 jklm	15982.69 ± 394.64 m	124.03 ± 2.94 jklm	108.01 ± 3.22 nop	104.01 ± 5.54 o	291.66 ± 11.42 mn	29.14 ± 0.82 q	85.29 ± 4.87 klm	1.19 ± 0.69 p	14.18 ± 0.86 t
V6	58.62 ± 0.85 gh	737.24 ± 41.98 jklm	15483.44 ± 259.18 m	116.21 ± 4.57 mno	121.08 ± 8.14 lmnop	73.23 ± 3.77 p	404.91 ± 12.58 ijklm	34.18 ± 0.59 no	68.5 ± 3.3 m	1.06 ± 0.62 p	12.87 ± 0.73 t
V7	53.46 ± 0.73 i	923.42 ± 57.32 efghi	17105.78 ± 232.24 klm	145.47 ± 3.11 ij	131.95 ± 1.17 jklm	114.86 ± 5.11 no	503.23 ± 12.65 cdefghi	69.72 ± 0.78 i	245.2 ± 9.75 d	164.92 ± 7.97 m	246.88 ± 0.81 f
V8	41.78 ± 0.93 l	692.43 ± 13.62 klmn	18493.08 ± 434.14 jkl	136.4 ± 1.66 jklm	123.5 ± 4.76 klmno	108.49 ± 9.77 no	233.74 ± 14.69 n	28.21 ± 0.94 q	111.73 ± 12.53 hij	0.63 ± 0.3 p	4.66 ± 0.27 u
V9	61 ± 0.92 gh	789.43 ± 17.75 hijk	24052.96 ± 390.23 def	186.84 ± 6.55 efg	101.58 ± 3.87 op	220.74 ± 9.86 i	375.81 ± 15.51 jklm	148.78 ± 0.5 a	423.58 ± 7.82 a	119.35 ± 7.07 n	417.55 ± 0.66 b
V10	56.52 ± 0.6 hi	1462.09 ± 81.87 a	18902.31 ± 649.27 jk	187.41 ± 6.77 efg	146.43 ± 9.66 j	344.28 ± 8.74 d	399.66 ± 32.8 ijklm	129.74 ± 0.89 c	315.06 ± 7.19 c	172.33 ± 9.27 m	330.02 ± 0.71 d
V11	67.33 ± 0.94 d	815.52 ± 18.37 ghijk	22365.6 ± 702.76 efgh	141.67 ± 7.38 ijk	204.83 ± 7.75 fgh	156.63 ± 1.51 kl	618.49 ± 73.29 bc	30.26 ± 0.53 pq	98.77 ± 1.02 ijkl	447.28 ± 5.68 ef	46.45 ± 0.53 n
V12	19.57 ± 0.64 p	439.34 ± 81.39 p	19749.78 ± 523.08 ij	91.5 ± 8.71 p	145.41 ± 2.05 jk	61.82 ± 1.59 p	433.51 ± 39.84 fghijkl	16.45 ± 0.91 t	83.41 ± 2.1 lm	3.86 ± 0.81 p	3.75 ± 0.1 u
V13	43.93 ± 0.61 kl	937.89 ± 10.5 efgh	24448 ± 421.18 de	219.04 ± 5.2 cd	275.15 ± 2.97 a	135.67 ± 6.06 lmn	500.25 ± 30.5 cdefghij	33.61 ± 0.57 no	94.13 ± 1.29 ijklm	364.18 ± 7.52 hi	136.49 ± 0.93 j
V14	53.39 ± 0.59 i	511.95 ± 11.9 op	19961.84 ± 297.23 ij	159.14 ± 1.86 hi	193.73 ± 4.35 ghi	202.84 ± 6.74 i	360.03 ± 33.2 klm	51.39 ± 0.58 k	115.94 ± 1.82 ghi	417.46 ± 5.44 g	118.25 ± 0.75 m
V15	45.76 ± 0.56 kl	604.11 ± 25.7 lmnop	24397.38 ± 229.7 de	207.46 ± 7.08 cde	210.09 ± 1.76 efg	280.14 ± 4.87 h	452.56 ± 40.73 efghijk	68.53 ± 0.53 ij	114.96 ± 4.48 ghi	465.82 ± 6.38 ef	138.94 ± 0.47 j
V16	42.3 ± 0.72 l	781.21 ± 22.61 hijkl	20469.48 ± 211.89 hij	140.6 ± 5.98 ijkl	138.05 ± 1.24 jkl	315.15 ± 7.4 ef	542.59 ± 10.47 cdefg	35.77 ± 0.98 no	110.06 ± 4.08 hijk	832.89 ± 6.55 a	24.92 ± 0.82 s
V17	53.98 ± 0.46 i	568.02 ± 45.05 mnop	24534.27 ± 582.67 d	245.25 ± 6.48 ab	111.79 ± 0.26 mnop	399.65 ± 4.87 c	589.19 ± 20.01 bcd	32.73 ± 0.35 nop	250.07 ± 7.08 d	328.56 ± 9.09 jk	130.01 ± 0.84 kl
V18	64.85 ± 0.83 de	756.39 ± 44.73 ijkl	26550.41 ± 774.63 c	129.26 ± 4.14 jklmn	218.7 ± 6.97 def	175.19 ± 9.01 jk	508.9 ± 11.17 cdefghi	32.54 ± 1.18 op	131.71 ± 4.59 fgh	247.8 ± 7.98 l	187.35 ± 0.85 i
V19	61.28 ± 0.5 fg	527.74 ± 16.57 nop	27029.96 ± 497.77 c	250.29 ± 8.6 a	174.7 ± 7.09 i	125.95 ± 1.84 mno	519.62 ± 13.8 cdefghi	83.58 ± 0.86 g	139.66 ± 5.78 fg	678.34 ± 9.58 c	37.08 ± 0.81 q
V20	41.42 ± 0.83 l	1037.96 ± 43.73 cdef	26626.09 ± 686.9 c	117.72 ± 6.99 lmno	227.09 ± 6.48 de	212.76 ± 7.95 i	538.29 ± 11.7 cdefgh	67.24 ± 0.89 ij	104.64 ± 3 ijkl	342.38 ± 8.2 ij	128.04 ± 0.89 l
V21	35.05 ± 2.13 m	490.24 ± 38.8 p	16559.06 ± 240.75 lm	106.65 ± 1.06 nop	131.93 ± 1.55 jklm	78.18 ± 9.83 p	483.8 ± 18.11 defghijk	25.29 ± 0.67 r	87.33 ± 1.68 jklm	1.4 ± 0.57 p	14.03 ± 0.75 t
V22	71.74 ± 0.8 c	931.79 ± 35.85 efghi	22657.42 ± 285.25 defg	132.53 ± 4.58 jklm	128.57 ± 2.95 jklmn	145.39 ± 6.87 lm	492.89 ± 22.79 defghij	40.62 ± 0.59 m	112.04 ± 6.51 hij	657.09 ± 6.99 c	22.77 ± 0.88 s
V23	50 ± 0.74 j	846.34 ± 32.6 ghijk	23048 ± 875.87 defg	204.77 ± 3.28 def	173.07 ± 5.22 i	333.76 ± 7.16 def	553.49 ± 14.94 cdef	44.58 ± 0.89 l	112.34 ± 8.7 hij	303.27 ± 8.54 k	131.93 ± 0.83 kl

V24	64.17 ± 0.58 ef	1137.99 ± 18.99 cd	23240.23 ± 326.71 defg	267.22 ± 8.31 a	185.58 ± 4.04 hi	514.85 ± 8.24 b	374.94 ± 30 jklm	137.16 ± 0.79 b	145.21 ± 6.68 f	757.67 ± 9.85 b	44.6 ± 0.56 no
V25	61.63 ± 0.63 fg	882.56 ± 17.3 fghij	18341.09 ± 126.58 jkl	169.19 ± 8.15 gh	149.56 ± 6.41 j	194.45 ± 7.56 ij	516.86 ± 26.77 cdefghi	43.01 ± 0.75 lm	138.74 ± 9.07 fg	326.63 ± 4.66 jk	227.8 ± 0.57 g
V26	93.02 ± 0.54 a	755.53 ± 26.15 ijkl	21703.89 ± 233.83 ghi	264.93 ± 2.87 a	264.88 ± 3.22 ab	212.1 ± 7.59 i	376.76 ± 15.46 jklm	86.48 ± 0.93 f	217.31 ± 7.83 e	316.55 ± 7.56 jk	257.3 ± 0.94 e
V27	73.71 ± 0.46 c	975.38 ± 25.88 defg	19817.68 ± 214.45 ij	188.19 ± 9.51 efg	130.88 ± 5.48 jklm	115.05 ± 4.83 no	412 ± 18.96 hijklm	67.81 ± 0.34 ij	219.06 ± 4.58 e	381.81 ± 7.12 hi	41 ± 0.28 p
V28	91.43 ± 0.39 a	1190.01 ± 27.94 bc	22735.08 ± 726.12 defg	184.61 ± 4.19 fg	213.67 ± 6.63 efg	340.86 ± 9.51 de	676 ± 28.58 b	114.31 ± 0.67 d	362.38 ± 9.58 b	567.29 ± 8.29 d	411.02 ± 0.74 c
V29	81.39 ± 0.94 b	1094.63 ± 39.18 cde	32065.83 ± 451.5 b	252.05 ± 2.32 a	235.81 ± 9.27 cd	865.31 ± 8.51 a	435 ± 37.76 fghijkl	65.89 ± 0.68 j	359.31 ± 8.84 b	435.45 ± 5.13 fg	198.11 ± 0.72 h
V30	90.57 ± 0.41 a	1337.32 ± 70.09 ab	22207.95 ± 260.71 fgh	227.7 ± 2.5 bc	251.32 ± 7.77 bc	312 ± 7.75 fg	393 ± 12.65 ijklm	77.79 ± 0.49 h	321.59 ± 6.31 c	440.72 ± 8.25 fg	439.87 ± 0.41 a

Note: Different lowercase letters in the table represents significant differences at the $p < 0.05$ level.

Table S4 Difference analysis of total polyphenols, total anthocyanins, vitamin C (VC), 2,2-diphenyl-1-picrylhydrazyl (DPPH), 2, 2'-azino-bis(3-ethylbenzothiazoline-6-sulfonic acid (ABTS), and ferric-reducing antioxidant power (FRAP) in 30 eggplant varieties.

Varieties	Total polyphenols	Total anthocyanins	VC	DPPH	ABTS	FRAP
	μg g^{-1} DW	μg g^{-1} DW	mg g^{-1}	%	%	U g^{-1}
V1	29204.58 ± 765.07 c	587.72 ± 6.58 gh	61.36 ± 0.99 m	16.54 ±0.29 ab	80.31 ± 1.30 ab	11.12 ± 0.94 b
V2	19593.43 ± 786.68 lmno	8.26 ± 0.74 q	66.52 ± 0.31 ghij	0.92 ± 0.08 k	57.57 ± 1.48 jk	1.80 ± 0.42 hij
V3	39474.98 ± 580.06 a	128.96 ± 3.99 p	73.63 ± 0.74 a	17.77 ± 1.56 a	86.86 ± 0.52 a	16.33 ± 1.18 a
V4	20252.24 ± 630.59 klm	29.14 ± 0.43 q	73.86 ± 0.21 a	2.26 ± 0.56 jk	58.57 ± 0.48 ijk	2.18 ± 0.85 hij
V5	17510.3 ±475.84 op	15.38 ± 0.71 q	69.95 ± 0.96 cde	0.61 ± 0.17 k	54.38 ± 1.89 k	1.04 ± 0.23 j
V6	17097.41 ± 327.86 p	13.93 ± 1.19 q	68.07 ± 0.73 efgh	0.55 ± 0.08 k	54.10 ± 0.93 k	0.94 ± 0.04 j
V7	19293.11 ± 179.42 mno	411.81 ± 7.42 o	69.25 ± 0.27 cdefg	1.10 ± 0.64 k	57.69 ± 4.68 jk	1.85 ± 0.12 hij
V8	19969.36 ± 440.66 klmn	5.28 ± 0.43 q	69.21 ± 0.28 cdefg	2.26 ± 0.42 jk	58.04 ± 1.00 ijk	1.94 ± 0.82 hij
V9	26360.71 ± 373.73 ef	536.91 ± 7.64 j	44.81 ± 0.21 o	8.18 ± 0.83 efgh	74.93 ± 0.62 bcdef	6.68 ± 1.03 cdef
V10	21943.5 ± 697.42 igk	502.35 ± 9.95 k	46.48 ± 0.74 o	3.54 ± 1.05 ijk	66.46 ± 1.34 fghij	3.25 ± 0.80 ghij
V11	24499.1 ± 794.95 fgh	493.73 ± 5.59 lk	62.58 ± 0.71 klm	4.95 ± 0.62 hij	69.55 ± 5.23 defg	4.12 ± 0.24 efghij
V12	21040.78 ± 570.93 ghlm	7.62 ± 0.77 q	64.87 ± 0.56 ijk	2.50 ± 0.13 jk	60.30 ±2.00 hijk	2.21 ± 0.43 hij
V13	26687.67 ± 442.37 def	500.67 ± 6.78 k	64.17 ±0.44 jkl	8.91 ± 0.61 ef	76.73 ± 1.02 bcde	6.98 ± 0.82 cdef
V14	21610.24 ± 266.15 gkl	535.71 ± 6.1 j	66.52 ± 0.78 ghij	3.42 ± 0.25 ijk	61.54 ± 1.21 ghijk	2.64 ± 0.04 hij
V15	26380.99 ± 225.42 ef	604.77 ± 5.97 g	67.14 ± 1.01 fghi	8.49 ± 0.93 efg	75.33 ± 4.50 bcdef	6.83 ± 0.40 cdef
V16	22575.22 ± 174.69 hig	857.81 ± 6.94 b	70.3 5± 0.27 cde	3.60 ± 0.62 ijk	66.88 ± 1.56 fghi	3.28 ± 0.24 ghij
V17	26784.95 ± 610.8 def	458.57 ± 8.8 nm	68.46 ± 0.23 defgh	9.46 ± 1.15 de	76.76 ± 1.43 bcde	7.14 ± 0.67 cdef
V18	28567.96 ± 843.69 cd	435.14 ± 8.77 no	66.18 ± 0.72 hij	11.11 ± 0.71 cde	77.95 ± 1.08 bcd	7.33 ± 1.39 cde
V19	28912.78 ± 518.23 c	715.42 ± 9.23 d	61.8 ± 0.97 lm	13.80 ± 0.84 bc	79.83 ± 1.80 ab	9.90 ± 1.60 bc
V20	28973.22 ± 710.93 c	470.42 ± 7.78 ml	51.96 ± 1.05 n	12.15 ± 0.49 cd	78.83 ± 0.77 abc	7.99 ± 0.48 cd
V21	17997.53 ± 205.25 nop	15.43 ± 1.14 q	51.61 ± 0.98 n	0.67 ± 0.21 k	56.68 ± 1.29 k	1.49 ± 0.59 ij
V22	24712.99 ± 293.69 efg	679.86 ± 6.5 e	71.5 ± 0.67 abc	5.31 ± 0.87 ghij	70.01 ± 1.35 cdefg	4.52 ± 0.98 efghi

V23	25366.35 ± 933.92 efg	435.19 ± 8.92 no	73.16 ± 0.05 ab	5.62 ± 0.82 fghij	70.03 ± 3.43 cdefg	4.63 ± 1.13 efghi
V24	26067.35 ± 317.79 efg	802.27 ± 9.58 c	69.33 ± 0.86 cdef	7.81 ± 1.43 efgh	74.30 ± 1.69 bcdef	5.11 ± 0.36 defgh
V25	20497.07 ± 172.12 gklm	554.43 ± 5.1 ij	70.96 ± 0.44 bcd	3.3 ± 0.43 ijk	60.77 ± 2.03 hijk	2.41 ± 0.53 hij
V26	23974.9 ± 227.03 ghi	573.85 ± 8.15 hj	63.4 ± 0.74 klm	4.52 ± 1.13 ij	68.19 ± 2.18 efgh	3.83 ± 0.41 fghij
V27	22000.47 ± 201.36 igk	422.81 ± 7.37 o	39.16 ± 0.63 p	3.48 ± 0.99 ijk	66.28 ± 0.91 fghij	2.89 ± 0.24 hij
V28	25909.16 ± 756.39 efg	978.32 ± 8.61 a	44.39 ± 0.44 o	8.12 ± 0.98 efgh	74.66 ± 0.69 bcdef	6.43 ± 0.25 defg
V29	35455.88 ± 489.58 b	633.57 ± 5.81 f	41.5 ± 0.29 p	16.91 ± 1.23 a	80.82 ± 1.86 ab	14.02 ± 1.51 a
V30	25219.95 ± 324.81 efg	880.58 ± 8.08 b	44.00 ± 0.55 o	6.11 ± 0.29 fghi	73.47 ± 1.57 bcdef	5.00 ± 0.78 defgh

Note: Different lowercase letters in the table represents significant differences at the $p < 0.05$ level.

TableS5 Compositional matrix of amino acids, antioxidants and antioxidant capacity of different Chinese eggplant varieties

Index	Components							
	PC1	PC2	PC3	PC4	PC5	PC6	PC7	PC8
Threonine	-0.666	0.575	0.101	0.199	0.230	0.190	0.058	0.061
Phenylalanine	-0.674	0.554	0.115	0.200	0.229	0.198	0.066	0.066
Leucine	-0.159	0.843	0.128	0.226	-0.113	-0.142	0.087	0.046
Isoleucine	0.209	0.380	0.783	0.054	-0.255	0.111	-0.164	-0.144
Asparagine	0.203	0.417	0.782	0.047	-0.209	0.089	-0.188	-0.125
Tryptophane	-0.379	0.748	-0.123	0.318	0.077	-0.095	0.068	0.220
Methionine	-0.323	0.787	-0.160	0.069	-0.061	-0.050	0.023	0.257
Proline	0.504	-0.291	0.163	0.363	-0.503	0.027	0.042	0.119
Valine	-0.161	0.566	0.114	-0.142	-0.250	0.398	0.295	-0.264
Tyrosine	-0.320	0.636	-0.033	0.477	0.204	-0.314	0.012	0.059
Cysteine	0.035	0.059	0.396	-0.303	0.333	-0.509	0.052	-0.266
Alanine	-0.014	0.185	0.434	-0.122	0.495	-0.098	-0.280	0.466
Glycine	0.528	0.109	0.407	-0.312	0.053	-0.269	-0.245	-0.217
Serine	0.272	0.450	0.644	0.055	0.163	0.014	0.215	0.061
Glutamic	-0.078	-0.125	0.071	-0.719	0.177	-0.179	0.186	0.389
Aspartic	0.629	0.591	0.034	-0.030	-0.155	-0.085	0.192	-0.098
Histidine	0.320	0.784	-0.045	-0.049	-0.184	0.088	0.157	-0.019
Cystine	-0.266	0.044	0.101	-0.106	-0.141	0.514	-0.507	0.105
Argnine	0.340	-0.404	0.239	-0.007	-0.418	-0.014	0.356	0.222
Glutamine	0.016	0.803	0.293	-0.053	0.130	0.026	-0.250	0.018
Total amino acids	0.444	0.420	0.330	-0.407	-0.210	-0.101	0.374	0.341
Gallic acid	0.583	0.522	-0.268	0.062	-0.265	-0.102	-0.074	0.141
Gentianic acid	0.377	0.491	-0.457	-0.004	0.065	0.074	-0.261	0.078

Chlorogenic acid	0.823	-0.260	0.116	0.204	0.345	0.227	0.109	-0.013
Caffeic acid	0.661	0.350	-0.250	0.107	0.083	-0.189	0.189	-0.130
Cynarin	0.524	0.609	-0.246	-0.301	-0.038	0.232	0.026	-0.123
p-Coumaric acid	0.539	-0.191	-0.531	0.066	0.104	-0.001	0.054	0.260
Ferulic acid	0.673	0.297	-0.183	0.065	0.118	-0.122	0.402	-0.128
Benzoic acid	0.501	-0.269	0.438	0.137	-0.324	0.056	-0.219	0.290
Rutin	0.441	0.563	-0.308	-0.021	0.328	0.065	-0.228	-0.146
Total polyphenol	0.860	-0.199	0.077	0.194	0.326	0.218	0.098	-0.006
Delphinidin-3-O-rutinoside chloride	0.635	-0.008	-0.048	0.238	-0.175	-0.543	-0.340	-0.010
Delphinidin chloride	0.408	0.428	-0.566	-0.220	-0.200	0.333	-0.078	0.090
Total anthocyanins	0.724	0.189	-0.300	0.100	-0.240	-0.307	-0.324	0.033
VC	-0.422	-0.331	0.149	0.671	-0.052	-0.065	0.196	0.044
DPPH	0.847	-0.188	0.228	0.183	0.273	0.229	0.015	0.067
ABTS	0.914	-0.167	0.034	0.125	0.225	0.157	-0.086	0.027
FRAP	0.830	-0.189	0.204	0.149	0.342	0.239	0.112	0.054

Note: Extraction Method: Principal Component Analysis. Eight components extracted.
